# More Than a Pore: A Current Perspective on the In Vivo Mode of Action of the Lipopeptide Antibiotic Daptomycin

**DOI:** 10.3390/antibiotics9010017

**Published:** 2020-01-03

**Authors:** Declan Alan Gray, Michaela Wenzel

**Affiliations:** 1 Newcastle University Biosciences Institute, Newcastle University, Newcastle upon Tyne NE2 4HH, UK; declan.gray@newcastle.ac.uk; 2 Division of Chemical Biology, Department of Biology and Biological Engineering, Chalmers University of Technology, 412 96 Gothenburg, Sweden

**Keywords:** daptomycin, lipopeptide antibiotic, mechanism of action, membrane domains, membrane fluidity

## Abstract

Daptomycin is a cyclic lipopeptide antibiotic, which was discovered in 1987 and entered the market in 2003. To date, it serves as last resort antibiotic to treat complicated skin infections, bacteremia, and right-sided endocarditis caused by Gram-positive pathogens, most prominently methicillin-resistant *Staphylococcus aureus*. Daptomycin was the last representative of a novel antibiotic class that was introduced to the clinic. It is also one of the few membrane-active compounds that can be applied systemically. While membrane-active antibiotics have long been limited to topical applications and were generally excluded from systemic drug development, they promise slower resistance development than many classical drugs that target single proteins. The success of daptomycin together with the emergence of more and more multi-resistant superbugs attracted renewed interest in this compound class. Studying daptomycin as a pioneering systemic membrane-active compound might help to pave the way for future membrane-targeting antibiotics. However, more than 30 years after its discovery, the exact mechanism of action of daptomycin is still debated. In particular, there is a prominent discrepancy between in vivo and in vitro studies. In this review, we discuss the current knowledge on the mechanism of daptomycin against Gram-positive bacteria and try to offer explanations for these conflicting observations.

## 1. Introduction

Daptomycin is a calcium-dependent cyclic lipopeptide, which was originally isolated in the 1980s from the Gram-positive soil actinomycete *Streptomyces roseosporus*. It was the first in class of a novel group of calcium-dependent, membrane-binding lipopeptides and was found to have impressive activity against Gram-positive, but not Gram-negative organisms [1,2]. Clinical studies were undertaken, however, it was found that high-dose treatment resulted in adverse effects, specifically myopathy [3], and as a result the antibiotic was shelved. Due to the drastic increase of antibiotic-resistant bacteria and the lack of sufficient novel antibiotic candidates daptomycin was revisited. Its side effects could be minimized through altering the dose regimen and it finally went on to receive approval from the U.S. food and drug administration (FDA) in 2003 [4]. Until the present day, the commercialization of daptomycin marks the last time that a new antibiotic class was introduced to the market.

Since daptomycin is active against antibiotic-resistant bacteria and to preserve the last effective antibiotics at disposal in the clinic, it was classified as a last resort antibiotic along with vancomycin and linezolid. Daptomycin is one of the few peptide antibiotics that can be administered systemically [5]. Daptomycin is used to treat skin infections, bacteremia, and right-sided endocarditis caused by Gram-positive bacteria, such as *Staphylococcus aureus*, both methicillin-susceptible and -resistant (MSSA and MRSA), as well as several *Streptococcus* and *Enterococcus* species [5]. Several more cyclic lipopeptide antibiotics have been discovered, but apart from the polymyxins and the antifungal echinocandins, daptomycin is the only one to currently have clinical approval [6].

Membrane-active antibiotics hold great promise for slower resistance development and have recently attracted renewed interest for drug development [7,8]. Daptomycin is the only systemically applied membrane-active antibiotic that is available for treatment of Gram-positive bacterial infections. Together with the anti-Gram-negative polymyxins and antifungal peptides like amphotericin B, daptomycin pioneered the systemic application of membrane-active anti-infectives. Learning from its successes and limitations will help to pave the way for the next generation of promising antimicrobial drugs. However, despite being well-established in the clinic, its exact mechanism is still debated. Intriguingly, there appears to be a crucial difference between its mechanism of action in model membrane systems and living bacterial cells. In this review we discuss the current knowledge on the mechanism of daptomycin against Gram-positive bacteria and try to explain the apparent in vivo–in vitro discrepancy in its behavior.

## 2. Structure and Oligomerization of Daptomycin

**Figure 1:**
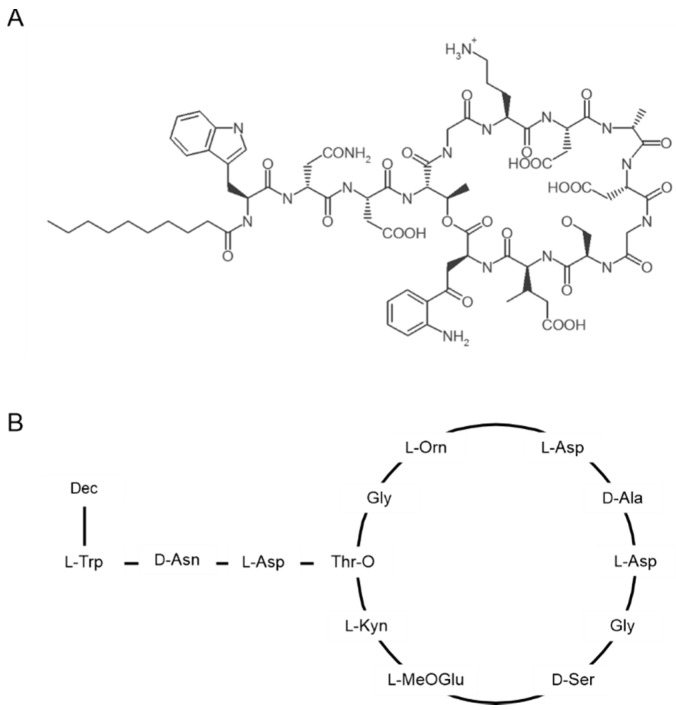
Structure of daptomycin. (**A**) Chemical structure. (**B**) Amino acid sequence. Dec: decanoyl chain, L-Orn: L-ornithine, L-MeOGlu: L-methylglutamic acid, L-Kyn: L-kynurenine.

Daptomycin is composed of 13 amino acids, 10 of which are arranged in a cyclic structure. The exocyclic tryptophan at position 1 carries a decanoyl fatty acid tail (Figure 1) [9,10]. The cyclic region of daptomycin contains several noncanonical and D-amino acids (kynurenine, ornithine, 3-methylglutamic acid, D-alanine, D-serine) [2]. Kynurenine and 3-methylglutamic acid have been shown to be crucial for daptomycin activity. Peptides carrying modifications at these positions exhibit up to five times higher minimal inhibitory concentrations (MICs) compared to unmodified daptomycin [11]. Another essential structural feature appears to be the ester bond between kynurenine and threonine [12]. Acidic residues are conserved in other calcium-dependent cyclic lipopeptides, for example friulimicin B and amphomycin A, emphasizing that complex formation with calcium and the resulting charge neutralization are essential features of this antibiotic class [13].

In contrast to other common lipopeptides like surfactin, polymyxins, or echinocandins, daptomycin has a negative net charge of −3 at pH 7 [14]. Its activity depends on the presence of Ca^2+^ ions, which reduce the negative charge of the peptide head groups and stimulate oligomerization of daptomycin [15,16,17,18]. The resulting daptomycin–calcium complex has a neutral net charge (2:3 daptomycin/Ca^2+^) [19]. Circular dichroism spectroscopy indicated that upon binding of calcium ions, daptomycin undergoes a structural transition that increases its amphipathicity [18]. NMR studies have suggested that the presence of calcium ions triggers the formation of daptomycin micelles, which are believed to facilitate its interaction with membranes [15]. Daptomycin micelles are also formed when other divalent cations, such as magnesium, are added, but higher ion concentrations are required and antimicrobial activity is lower [20]. The Ca^2+^–daptomycin complex has an increased affinity for negatively charged phospholipids including phosphatidylglycerol (PG). Binding to PG induces a second conformational change of the daptomycin complex allowing membrane insertion and assembly of its final, active conformation [21]. However, other studies have challenged these findings and suggested that daptomycin does not undergo a structural transition upon binding Ca^2+^ prior to membrane binding. Instead, there may only be two states of daptomycin, free and membrane-bound [19].

Despite these conflicting observations on the exact structural transitions of daptomycin, its PG-dependent oligomerization has been observed in model membrane systems, isolated bacterial membranes, and bacterial cells [21,22,23], and it was shown that it forms distinct daptomycin–PG domains in vitro [24]. PG is prevalent in the membranes of bacteria and is thought to promote the selectivity of daptomycin for bacterial over mammalian membranes [18,25]. PG has been identified to be the docking molecule of daptomycin and is essential for its activity (see also Section 8) [21,25,26,27,28,29,30,31,32,33,34,35,36,37,38,39,40,41,42,43,44]. Thus, daptomycin does not bind to PG-free membranes in vitro [22] and the presence of PG is a prerequisite for its antibacterial activity [25,45]. PG is particularly abundant in Gram-positive cell membranes [46] and indeed daptomycin binds to the membrane of Gram-positive, but not Gram-negative bacteria, which has been proven in vitro using model membrane systems and in vivo using *Escherichia coli* protoplasts [47,48].

Fluorescence resonance energy transfer (FRET) experiments suggested that the calcium–daptomycin complex in the membrane consists of 6–7 subunits. However, FRET cannot detect the presence of a second 6–7-mer that could possibly sit in the inner membrane leaflet. Since it was unknown at the time if daptomycin could flip to the inner leaflet, it was proposed that the active complex could consist of 12–14 daptomycin molecules instead [22]. This is still a controversial question and later studies have suggested one 8-mer per leaflet [49]. Another study found that flipping is inhibited by the presence of cardiolipin [50]. However, another study found no evidence for translocation of the lipopeptide over the membrane [24]. Thus, flipping of daptomycin to the inner membrane leaflet is still debated and whether it could happen in bacterial cells remains entirely unknown.

## 3. Mechanism of Action in Model Membranes

**Figure 2:**
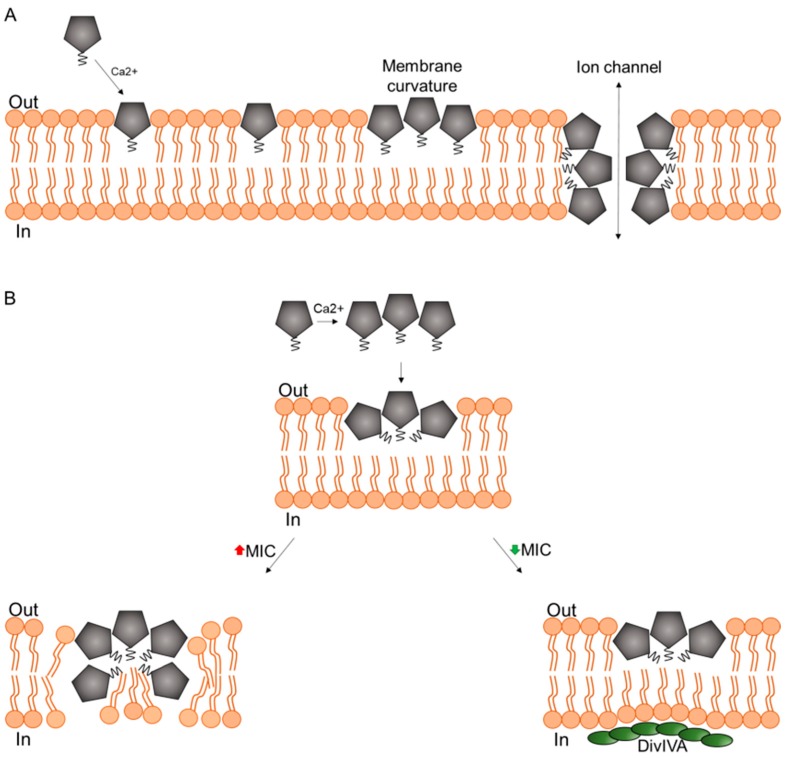
Pore formation model of daptomycin action. (**A**) Original model of pore formation by daptomycin. Adapted from [63]. (**B**) Dual stage model of daptomycin action as proposed by [64].

A multitude of model membrane studies have been conducted with daptomycin and most of them agree that it binds to PG-containing membranes in a calcium-dependent manner, and subsequently causes leakage of solutes through the lipid bilayer. In vitro studies on the mechanism of daptomycin have been the subject of other extensive reviews [51,52]. Since the focus of this review is on the in vivo mechanism, we will briefly summarize some key examples that have contributed to the wide-spread pore formation model of daptomycin action (Figure 2A).

Daptomycin has been shown to bind and insert into model membranes, inserting deeper into membranes containing PG. This interaction is accompanied by membrane leakage as measured through calcein release [18]. Studies on liposomes made of 3:1 DMPC/DMPG (1,2-dimyristoyl-sn-glycero-3-phosphocholine/1,2-dimyristoyl-sn-glycero-3-phosphoglycerol) have systematically analyzed the permeability of daptomycin pores [53]. It was found that permeability was highest for Na^+^, K^+^, and other alkali metal ions, followed by Mg^2+^, and organic cations, while no increased permeability was observed for anions. The study concluded that influx of sodium ions leading to membrane depolarization is likely the mechanism of action of daptomycin against bacteria. This hypothesis was later tested in *Bacillus subtilis*, but no sodium influx was observed [54]. A later study used different lipid mixtures, including 3:1 DMPC/DMPG and 1:1 POPC/POPG (1-palmitoyl-2-oleoyl-sn-glycero-3-phosphocholine/1-palmitoyl-2-oleoyl-sn-glycero-3-phosphoglycerol), and found that daptomycin pores are likely selective for potassium ions [55]. In fact, some studies have observed potassium leakage from bacterial cells [56,57,58]. Several studies supported the model of a more or less organized daptomycin pore. For example, Zhang et al. found that daptomycin translocates to the inner membrane leaflet and concluded that it forms a membrane-spanning pore. This was supported by the finding that cardiolipin not only inhibits the translocation of daptomycin to the inner membrane leaflet, but also diminishes the bilayer permeabilization [50]. However, other studies have suggested that daptomycin rather induces membrane permeability by deforming the membrane, clustering membrane lipids, and inducing only transient membrane leakage, or even no leakage at all [24,49,59]. These vastly conflicting observations are most likely due to differences in model membrane composition and peptide concentration [49,60,61,62]. Thus, studies have found that the ability of daptomycin to permeabilize model membranes does not only depend on the presence of PG, but also on fatty acid chain length and membrane fluidity [49,57,62].

## 4. Pore Formation In Vivo

Several studies have been performed on living bacteria in vivo, some of which seemed to support the pore formation model of daptomycin action while others seemed not to. However, upon closer inspection of the individual experimental conditions in these studies, it becomes clear that there are two key factors that need to be considered in this central question: peptide concentration and treatment time. In fact, there is a remarkable consensus in the literature that membrane depolarization and ion leakage only occur at high concentrations, typically much higher than the MIC and often bacteriolytic, and prolonged treatment times of at least 30 to 60 min. This is in sharp contrast to typical pore-forming molecules, which cause near-instantaneous depolarization and intracellular content leakage at their MICs [65,66,67,68,69,70], or even slower carrier ionophores, which still achieve 100% ion leakage within a few minutes [66].

These observations have been consistently made in different microorganisms. In *S. aureus*, daptomycin has been shown to be bactericidal without causing cell lysis and did not show calcein release or uptake of the membrane-impermeable DNA-binding dye ToPro3, even at very high concentrations up to 5–10× MICs and up to 60 min treatment time [71]. Similarly, *S. aureus* cells were negative for BacLight membrane permeability staining (based on influx of the membrane-impermeable DNA-binding fluorescence dye propidium iodide), ATP leakage, and release of beta-galactosidase after 10 min treatment with 4× MIC. Importantly, while leakage of K^+^, Mg^2+^, and ATP as well as membrane depolarization, were observed on a longer time scale (up to 2 h), all of these effects only set in after ≥99% of the cell population were already dead (10–20 min), demonstrating that they are consequences of cellular decay rather than the basis for bacterial killing [72]. The same behavior was observed by Jung et al., who showed that depolarization follows the killing of *S. aureus*. Moreover, maximum depolarization was only achieved after 90 min treatment with 10× MIC, since lower concentrations had no effect on the membrane potential [18]. Silverman et al. also showed that membrane depolarization correlates with cell death, yet it seemed to occur concomitantly. This paper has often been referred to as proof for pore formation, yet it clearly showed slow, gradual loss of membrane potential with maximum depolarization only seen after 30–60 min of treatment with 8× MIC [56]. Similarly, potassium release experiments showed that next to no potassium release was observed at concentrations that were sufficient to kill 90% of the cell population [56]. Similarly, Mensa et al. reported only partial depolarization of *S. aureus* treated with 4× MIC for 30 min [73]. Even when cultures were treated with an overkill of 25–100× MIC, it still took about 5 min to achieve maximum membrane depolarization and influx of the membrane-impermeable DNA-binding fluorescence dye Sytox green [74]. Similar results were found at 80× MIC using BacLight as a reporter [57]. In line with these findings on *S. aureus*, depolarization in *Staphylococcus epidermidis* occurred at 2–4× MIC after 60 min of treatment and it took 16× MIC to observe depolarization at only 15 min. Depolarization and killing kinetics were comparable to *S. aureus* and it took 60 min of treatment with 20–80× MIC to observe about 40% of BacLight-positive cells [75]. In *Bacillus anthracis*, no ToPro3 uptake was observed and depolarization was concentration-dependently achieved within 30 min. However, even 5× MIC did not result in complete depolarization. At this concentration, relatively rapid (2–5 min) efflux of potassium and influx of sodium ions were observed. However, potassium efflux was only 60% of the release measured with the carrier ionophore valinomycin [58]. In *B. subtilis*, ATP leakage experiments showed that it takes 5× MIC and treatment times of 60–120 min to achieve about 80% loss of intracellular ATP [76]. Another paper showed concentration and calcium-dependent influx of propidium iodide into *B. subtilis* cells at time frames of >120–30 min, yet it is not clear to what MIC multiples these concentrations correspond [77]. A different study showed that *B. subtilis* cells are BacLight-negative at 2× MIC [54]. The same paper showed that neither ion leakage (15 min) nor cell lysis occur at inhibitory concentrations (2× MIC). Under these conditions, depolarization was slow and incomplete (40 min, about 50% maximum). Bacteriolytic concentrations did achieve full depolarization, yet this still took 30 min [54].

While some of these results are still repeatedly used as proof for in vivo pore formation by daptomycin, these studies are surprisingly consistent in supporting the notion that daptomycin does not primarily act as a pore-forming molecule.

## 5. Is It Cell Wall Synthesis after All?

If daptomycin does not form pores in vivo, what is its mechanism of action then? Some of the very first studies on daptomycin suggested that it inhibits the synthesis of lipoteichoic acids (LTAs), a major constituent of the Gram-positive cell wall. This was concluded from the observation that incorporation of radioactive precursors into LTAs of *S. aureus* and *Enterococcus faecium* was strongly inhibited (about 80%–90%). These effects were already observed at 1× MIC and 10–20 min incubation time. While precursor incorporation into lipids and peptidoglycan was also inhibited by about 50%, no major effects were observed on DNA, RNA, and protein synthesis [78]. LTAs are bound to the cytoplasmic membrane with a lipid anchor and it was further observed that radioactively-labeled daptomycin specifically binds to cytoplasmic membrane fractions [78]. Finally, accumulation of an LTA precursor molecule and depletion of the following LTA intermediates by daptomycin further corroborated the LTA inhibition hypothesis [79]. However, this hypothesis was refuted by Laganas et al., who performed kinetic and dose-response experiments showing no specificity of daptomycin for the inhibition of the synthesis of LTA over other macromolecules in both *S. aureus* and *Enterococcus faecalis* [80].

Daptomycin did not bind to cell wall fractions [78] and was able to kill *Enterococcus faecium* protoplasts [81]. Thus, it was concluded that peptidoglycan cannot be its target. However, it could still inhibit peptidoglycan synthesis by interacting with the membrane-bound precursor molecule lipid II. This was put forward by a study showing strong inhibition of cell wall precursor incorporation and accumulation of the lipid II precursors Uridine diphosphate N-acetylglucosamine (UDP-GlcNAc) and Uridine diphosphate N-acetylmuramic acid (UDP-MurNAc) pentapeptides in *S. aureus*, *Bacillus megaterium*, and cell-free systems [9,82]. This was further supported by scanning electron microscopy images of *S. aureus* and *E. faecalis* showing massive cell wall distortions [83]. However, the lipid II hypothesis was also rejected when it turned out that the addition of lipid II precursors did not antagonize daptomycin activity [84] and that it did neither bind to lipid II nor inhibited lipid II synthesis in vitro [85]. Further evidence against the lipid II hypothesis was provided by the observation that daptomycin is active against cell wall-less *Mycoplasma orale* and *Mycoplasma arginini* [86], cell wall-less *B. subtilis* L-forms [87], and non-growing *S. aureus* persister cells [88]. In contrast, daptomycin was inactive against *E. coli* protoplasts, suggesting that it is not the outer membrane barrier that renders it ineffective, but that the target of daptomycin is actually absent from Gram-negative bacteria. This was confirmed for *Pseudomonas aeruginosa*, *Enterobacter cloacae*, *Klebsiella pneumonaie*, *Moracella catarrhalis*, and *Salmonella typhimurium* [45] and further supported the notion that the target of daptomycin cannot be lipid II.

Despite this clear evidence that daptomycin does not directly inhibit peptidoglycan synthesis, studies continued to find cell wall-related phenotypes and stress response profiling showed induction of cell wall stress stimulons. Thus, daptomycin acts synergistically with beta-lactam antibiotics [89,90,91] and proteomic studies found induction of cell wall stress response proteins in both *B. subtilis* and *S. aureus* [54,66,92,93]. Likewise, cell wall stress stimulons were also found to be upregulated in transcriptomic datasets [43,92,94] and promotor activation studies [87,95].

## 6. A New In Vivo Mode of Action Model

**Figure 3:**
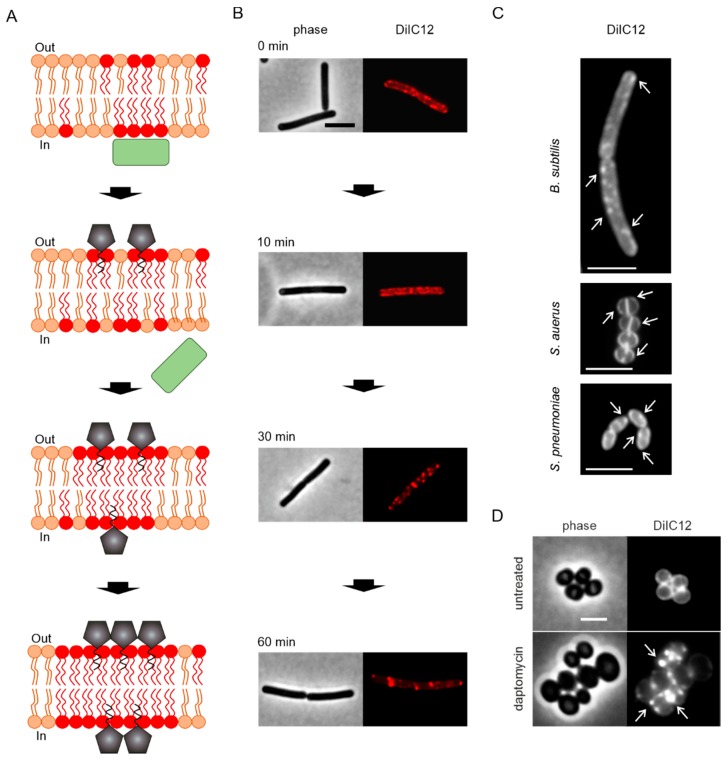
New in vivo model of daptomycin action. (**A**) Molecular model of membrane interaction and protein delocalization. Adapted from [54]. (**B**) Corresponding microscopy pictures showing phase contrast and DiIC12 fluorescence. DiIC12 is a fluid lipid domain dye that visualizes regions of increased fluidity (RIFs) in both Gram-positive and Gram-negative bacteria [96,97]. Images were previously published in [54]. (**C**) Fluid membrane domains (indicated by arrows) stained with DiIC12 in different Gram-positive bacteria: *B. subtilis*, *S. aureus*, and *Streptococcus pneumoniae*. Images were previously published in [98]. (**D**) Accumulation of fluid membrane domains (arrows) by daptomycin in *S. aureus*. Scale bars: 2 µm.

For a long time the question of whether daptomycin inhibits cell wall synthesis or not has remained a conundrum, but a handful of recent in vivo and in vitro studies cleared up much of the mist around this long-standing mystery. Pogliano et al. discovered that daptomycin causes patches in the cell membrane of *B. subtilis* that coincide with cell shape deformations and co-localize with reporters for cell wall synthesis, namely fluorescently-labeled vancomycin, binding lipid II, and bocillin, a fluorescently-labeled version of penicillin. The authors concluded that daptomycin causes a change in membrane organization that leads to misdirection of cell wall biogenesis and proposed a revised model of its mechanism on bacterial membranes (Figure 2B) [64]. This model was well in line with the induction of cell wall stress stimulons in normally growing *B. subtilis* cells, but not in cell wall-less L-forms [87]. Müller et al. then proceeded to study the effects of daptomycin on *B. subtilis* cells and found that the lipopeptide preferentially inserts into fluid membrane microdomains, so-called RIFs (regions of increased fluidity) [54]. These RIFs are organized by the MreB protein and harbor the lateral cell wall synthesis machinery [54,96]. Daptomycin causes an immediate rigidification of the cell membrane, including RIFs, causing peripheral membrane proteins to lose contact to these domains, most importantly the essential lipid II synthase MurG (Figure 3A,B). These observations could finally explain why daptomycin causes a similar phenotype and stress response to lipid II-binding antibiotics but does not bind to lipid II or inhibit its synthesis in vitro [85].

Müller et al. observed the same membrane patches previously described by Pogliano et al. and showed that they correspond to RIFs that were rigidified and fused together by daptomycin (Figure 3B). RIFs were originally defined as fluid membrane microdomains that coordinate lateral cell wall synthesis and are organized by MreB and have been observed in both Gram-positive and Gram-negative bacteria [54,96,97]. However, the main targets of daptomycin therapy are Gram-positive cocci, which neither possess lateral cell wall synthesis, nor MreB. They do, however, possess fluid membrane microdomains that can be visualized with the same fluid lipid domain dye (Figure 3C). In fact, daptomycin also fuses these domains to similar membrane patches in *S. aureus* cells (Figure 3D). It was later shown that these sites, which are often accompanied by cell shape deformations in *B. subtilis* [64], indeed showed aberrant peptidoglycan structures [99], supporting the models put forward by Pogliano et al. and Müller et al.

These rearrangements in the cell membrane also affected membrane proteins other than MurG. Thus, the phospholipid synthase PlsX also co-localized with RIFs and was displaced by daptomycin as fast as MurG [54]. This might explain why both membrane and cell wall synthesis were originally observed to be impaired by daptomycin [78]. Pogliano et al. found the cell division-regulating protein DivIVA to be mislocalized to these sites, providing an explanation for previously observed septal defects and elongated cells [71,78]. This mislocalization was later shown to be an artifact caused by dimerization of green-fluorescent protein (GFP), but DivIVA nonetheless turned out to be affected by daptomycin. Using a monomeric version of GFP, it was shown that the protein is sensitive to dissipation of the membrane potential and loses its membrane binding upon prolonged (≥30 min) treatment with daptomycin [54]. This membrane potential dependency has been observed as well for other proteins involved in cell division, including FtsA and MinD, and for the MreB protein [100]. Indeed, these proteins also lost their membrane binding upon prolonged daptomycin treatment, explaining the cell division defects observed in earlier studies [71,78]. Other proteins, including integral membrane proteins interacting with MreB or MurG, were not affected by daptomycin-induced changes in membrane fluidity and architecture [54]. 

**Figure 4:**
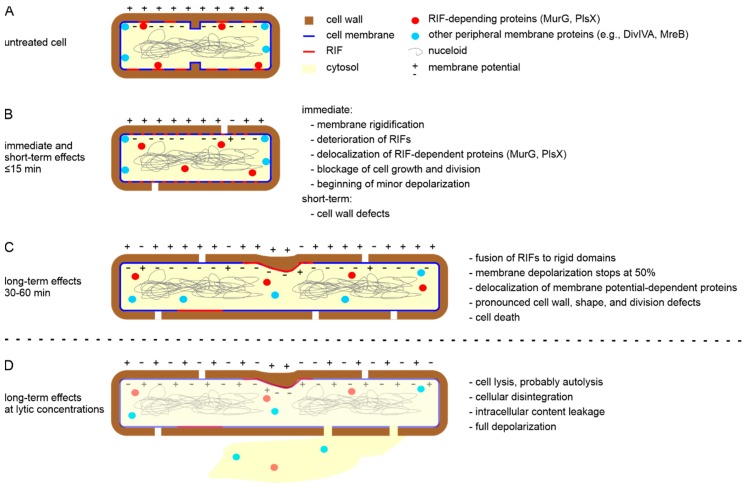
Effects of daptomycin on *B. subtilis* cells. (**A**) Untreated cell. (**B**) Short-term effects of daptomycin (0–15 min) at bactericidal concentrations. (**C**) Long-term effects of daptomycin (30–60 min) at bactericidal concentrations. (**D**) Long-term effects of daptomycin (30–60 min) at bacteriolytic concentrations.

Importantly, while effects on membrane permeability were typically observed at supra-MICs and longer treatment times (see Section 4), membrane rigidification and displacement of MurG were observed immediately at 1× MIC [54]. Figure 4 sums up the sequence of events observed in *B. subtilis* cells at inhibitory concentrations (1–2× MIC) [54,64,99].

**Figure 5:**
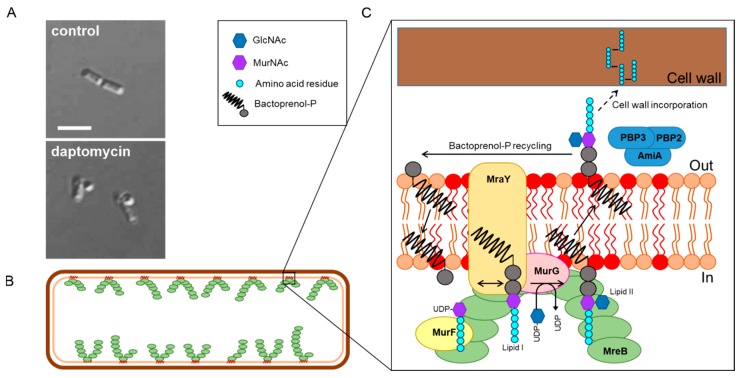
Coordination of cell wall synthesis in RIFs. (**A**) Inhibition of cell wall synthesis by daptomycin. Fixation of *B. subtilis* cells in a 1:3 mixture of acetic acid and methanol leads to extraction of the protoplast through breaches in the peptidoglycan layer. This is visible as blebs on the cell surface [66,111]. (**B**) MreB filaments orchestrate RIFs and drive them forward in a spiraling motion to regulate lateral cell wall synthesis. (**C**) The cell wall synthesis machinery localizes in RIFs.

Immediately after daptomycin addition, membrane rigidification and disruption of RIFs sets in, which is accompanied by displacement of RIF-bound proteins, MurG and PlsX (≤2 min). Depolarization sets in but progresses very slowly. Cell growth and division come to a halt. These events are followed by impairment of cell wall synthesis (10–15 min, Figure 5A). Depolarization reaches a plateau at 50% after 40 min. This is accompanied by displacement of membrane potential-sensitive peripheral membrane proteins. Between 30 and 60 min RIFs have fused to rigidified membrane patches and pronounced cell wall, shape, and division effects become visible. At supra MICs, cells lyse, probably caused by deregulation of cell wall-autolytic enzymes [101,102]. Under these conditions, cells fully depolarize after 30 min and cellular disintegration leads to intracellular content leakage [54,77]. However, cell lysis is not a requirement for the bactericidal activity of daptomycin [71], suggesting that its effects on cell envelope homoeostasis are sufficient to kill bacteria.

Following these studies, Lee et al. set out to test these observations in model membranes [59]. Using giant unilamellar vesicle (GUV) studies, the authors confirmed that daptomycin has a preference for the liquid crystalline over gel phase and that daptomycin binding is reduced by the membrane-stiffening cholesterol. They further found that ion leakage by daptomycin is transient and only occurs upon initial binding to the lipid bilayer. Additionally, a certain threshold concentration is required to cause ion leakage, which is well in line with the in vivo data. Moreover, GUV studies by Kreutzberger et al. demonstrated that daptomycin forms microscopically visible membrane domains with PG [24].

## 7. More to Discover

Does daptomycin form domains with fluid lipid domains (RIFs) or rather with PG domains? The answer is probably both, because it is very likely that RIFs are also enriched in PG [103]. Since daptomycin has an affinity to both negatively charged PG and higher fluidity [24,59], this explains why it localizes to these domains [54].

It has been shown that *B. subtilis* possesses so-called lipid spirals that are enriched in anionic phospholipids [103]. This phospholipid is very likely PG and not cardiolipin [103], which would fit well with the observation that PG is needed for, and cardiolipin counteracts, daptomycin action [22,25,50]. Lipid spirals depend on active lipid II synthesis by MurG [104]. MurG is coupled to MreB, which drives the lipid spirals around the cell to orchestrate lateral peptidoglycan synthesis [105,106]. Newer studies have found that rather than forming an actual spiral, MreB forms short filaments that align themselves along the greatest principal membrane curvature to drive cell wall synthesis in a spiraling movement along the lateral axis of the cell [107]. These observations put forward a model, in which RIFs are fluid membrane microdomains enriched in PG that contain the lateral cell wall synthesis machinery and are organized by MreB (Figure 5).

While it was first assumed that MreB establishes RIFs, it is now known that it is needed for RIF distribution at the long axis of the cell, but not for their generation [67]. Rather, MreB filament formation and dynamics depend on the presence of lipid II [108], explaining why MurG depletion diminishes these domains [104]. It now emerges that lipid II itself is intimately linked to the higher fluidity of RIFs, which have been shown to harbor the cell wall precursor [64,104]. Lipid II possesses an undecaprenyl membrane anchor, which is bound to the cell wall sugar building block with a pyrophosphate group. This undecaprenyl lipid tail with the large sugar ‘head group’ is prone to increase membrane disorder. It is therefore likely that lipid II (i) thermodynamically favors the fluid phase and (ii) generates additional membrane disorder aggravating local membrane fluidity. Indeed, the disorder-increasing effect of lipid II has been experimentally observed [109,110]. Binding of daptomycin to these complex and highly organized domains, mediated by (i) their higher fluidity and (ii) higher PG content, likely disturbs multiple protein–protein and protein–lipid II interactions that are essential for peptidoglycan synthesis. Thus, it comes as no surprise that this lipopeptide exerts cell wall stress responses and causes cell wall inhibition phenotypes in various assays.

A similar behavior was observed for human beta-defensin 1, which was shown to display a low affinity to lipid II, probably mainly due to electrostatic interactions of the positively charged peptide with the negatively charged pyrophosphate group. This leads to preferential localization of the defensin to sites of active peptidoglycan synthesis, which was believed to cause disruption of the highly coordinated cell wall-synthetic machinery [112]. This ‘sand in the gearbox’ principle is probably also applicable to daptomycin. Moreover, since the calcium–daptomycin complex behaves similarly to cationic antimicrobial peptides in that it binds to negatively charged PG and is repelled by positively charged lysyl-PG (see Section 8) [113], it is possible that a similar low-affinity binding of daptomycin to lipid II and/or undecaprenyl phosphate, which so far defied detection, could contribute to its attraction to RIFs and inhibition of cell wall synthesis.

Undecaprenyl phosphate is not only the carrier molecule for lipid II but also is used to translocate wall teichoic acids (WTAs) over the membrane [114]. It is therefore likely that WTA synthesis is also localized in RIFs. This is consistent with WTA-synthesizing enzymes localizing in a similar helical pattern [115]. While it is not exactly known where LTA synthesis is organized in *B. subtilis*, it would make sense that it likewise localizes where new peptidoglycan material is incorporated into the cell. Thus, it could be speculated that daptomycin does not only disrupt peptidoglycan and lipid synthesis, but also teichoic acid synthesis through its interaction with RIFs, which could explain the very early observations on inhibition of these pathways [78].

It has been shown that daptomycin triggers autolysis in *B. subtilis* [101] and reduced autolytic enzyme activity is a key feature in the transition from vancomycin-susceptible (VSSA) to intermediate (VISA) *S. aureus* phenotypes, which show cross-resistance with daptomycin [102]. The same reduction of autolysin activity was observed in daptomycin-resistant laboratory strains of *S. aureus* [116]. An interesting observation pertaining to autolysis was made for another antibiotic class, the theta-defensins. These peptides trigger autolysis by binding LTAs in *S. aureus* [117]. In this organism, the major autolysin Atl is controlled by an interplay of WTAs and LTAs. WTAs in the old cell wall repel Atl and force it to the division site, where it binds to LTAs and exerts its autolytic activity to selectively lyse the peptidoglycan crosswall between daughter cells [118,119]. Upon binding to LTA, theta-defensins release Atl causing uncontrolled digestion of cell wall peptidoglycan and thus cell lysis [117]. Considering the findings for daptomycin concerning autolysis [101,102] and LTA inhibition [78,79,81,120], it will be interesting to examine whether daptomycin induces autolysis through a similar mechanism.

## 8. Lessons from Daptomycin Resistance

Reduced autolysis is not the only resistance mechanism that can give insight into the mechanism of action of daptomycin. In fact, a long list of genetic factors that reduce its activity have been identified (Table 1). Most of these affect membrane and cell wall homoeostasis and support the newest in vivo model of daptomycin action (Figure 3).

Probably the most well-known daptomycin resistance mechanisms are related to phospholipid composition. Gram-positive bacteria like *S. aureus* possess three major phospholipids: PG, lysyl-PG, and cardiolipin [121]. One common daptomycin resistance mechanism is reduction of the overall PG content by reducing the activity of the PG synthase PgsA. This mechanism reduces the possible binding sites for daptomycin and was found in both *B. subtilis* and *S. aureus* [25,43,44]. Streptomycetes like *S. roseosporus* generally have a low PG content, which might explain how the producer strain copes with daptomycin [51,122]. A similar strategy is lysinylation of PG, resulting in reduction of negatively charged PG in favor of positively charged lysyl-PG. This does not only reduce the overall content of PG in the membrane, it also alters the net charge of the cell surface, possibly repelling the calcium–daptomycin complex [113]. This mechanism is mediated by MprF and constitutes one of the best characterized daptomycin resistance mechanisms in *S. aureus* [26,27,28,29,30,31,32,33,34,35,36,37,38,39,40,41,42]. Increased cardiolipin content is another common resistance mechanism and has been described in *S. aureus*, *E. faecalis*, and *E. faecium* [44,45,46,47,48,49,50,51,52,53,54,55,56,57,58,59,60,61,62,63,64,65,66,67,68,69,70,71,72,73,74,75,76,77,78,79,80,81,82,83,84,85,86,87,88,89,90,91,92,93,94,95,96,97,98,99,100,101,102,103,104,105,106,107,108,109,110,111,112,113,114,115,116,117,118,119,120,121,122,123,124,125,126,127,128,129,130,131,132,133]. Cardiolipin synthesis consumes two PG molecules per cardiolipin and thus also contributes to reduction of the PG content [134]. However, cardiolipin seems to additionally counteract daptomycin activity, possibly by increasing membrane stiffness [50,135]. These three resistance mechanisms are related to the balance of the major phospholipid species and confirm the importance of PG as a docking molecule for daptomycin, as well as the importance of the net charge of the membrane and membrane fluidity. 

A recent paper described another PG-related mechanism of how *S. aureus* populations can cope with daptomycin, namely by phospholipid shedding [136]. Pader et al. showed that *S. aureus* reacts to daptomycin by shedding lipids into the surrounding medium and that free PG outside the cells can sequester and inactivate daptomycin. However, wild type cells also secrete small peptide cytolysins that act as surfactants and impair this mechanism. Mutants defective in the secretion of these molecules effectively inactivate daptomycin and thus protect themselves and, in mixed populations, also wild type cells from its activity [136]. This discovery not only underlines the importance of PG as a docking molecule for daptomycin but also shows how bacteria can turn their weak spot into an effective resistance strategy.

**Table 1:** Mutations that confer daptomycin resistance.

Species	Mutated Gene	Gene Function	References
*B. subtilis*	*pgsA*	PG synthase	[25,43]
	*mprF*	lysinylation of PG to lysyl-PG	[43]
	*liaSR*	cell envelope stress response	[25,43,92,137]
*S. aureus*	*pgsA*	PG synthase	[44]
	*mprF*	lysinylation of PG to lysyl-PG	[26,42]
	*cls*	cardiolipin synthase	[44,123,124,125,126]
	*walKR*	cell wall and membrane homeostasis	[37,138,139,140]
	*dtlABCD*	D-analylation of cell wall teichoic acids	[141,142,143,144,145,146]
	*graRS*	upregulation of *dtl* operon	[116,147]
	*vraSR*	cell envelope stress response	[52,148]
	*SAOUHSC_00362*	hypothetical lipoprotein	[149]
	*SAOUHSC_02441*	alkaline shock protein	[149]
*E. faecalis*	*cls*	cardiolipin synthase	[127,128,129]
	*gdpD*	glycerophosphoryl diester phosphodiesterase	[127,128]
	*liaSR*	cell envelope stress response	[127,128,150]
*E. faecium*	*cls*	cardiolipin synthase	[127,130,131,132,133]
	*walKR*	cell wall and membrane homeostasis	[133]
	*liaSR*	cell envelope stress response	[127,130,151,152,153]

Two more resistance mechanisms are involved in membrane remodeling, yet how exactly they confer daptomycin resistance is less well characterized. Mutations in gdpG encoding a glycerophosphoryl diester phosphodiesterase, confer high levels of daptomycin resistance in *E. faecalis* when they occur together with mutations in liaSR. GdpG is involved in glycerol turnover for phospholipid synthesis and thus also appears to be related to membrane adaptation [134]. The WalKR two-component system has many different functions in cell wall and membrane homoeostasis as well as cell division [154,155]. Interestingly, WalKR is involved in regulation of autolysis in *B. subtilis* [156] and in the regulation of membrane fluidity in *S. pneumoniae* [157]. Moreover, the lipid desaturase, Des, which reduces membrane fluidity in *B. subtilis* [158], was found to play a role for daptomycin susceptibility in this organism [43]. Thus, adaptation of membrane fluidity seems to play a key role in daptomycin resistance along with reducing PG content.

Another well-characterized daptomycin resistance mechanism is mediated by mutations in the dlt operon or in graRS encoding a two-component system that is involved in its regulation. These mutations enhance D-alanylation of WTAs, a well-known strategy to decrease the negative net charge of the cell wall to repel positively charged molecules such as antimicrobial peptides [159]. It has been argued that daptomycin, when in complex with Ca^2+^, might behave similarly to cationic antimicrobial peptides, which might explain why this resistance mechanism is successful [142].

The last common group of genes that confer daptomycin resistance are stress response regulators. LiaSR is a two-component system that is involved in adaptation to cell envelope stress. It has been well-studied in *B. subtilis* [137,160,161,162] and was shown to react specifically to inhibition of membrane-bound steps of peptidoglycan synthesis [66]. Yet, its exact function is still not known. Homologues exist in a wide range of pathogens, including *S. aureus* (VraSR), *E. faecalis*, and *E. faecium*. It is involved in daptomycin resistance in all four organisms [134]. This underlines its critical role in daptomycin resistance and supports the RIF-centered in vivo model of daptomycin action.

## 9. Closing the Gap between In Vitro and In Vivo

Does that mean that all studies proposing membrane pores are wrong? This is certainly not the case. Pore formation, one way or another, undoubtedly happens in in vitro model membrane systems and, if the concentration is high enough, daptomycin is able to destroy bacterial cells causing leakage of intracellular content. However, we have to distinguish what daptomycin is able to do under certain conditions and what its antibacterial mechanism of action is at around its minimal bactericidal concentration. We also have to take into account what its direct mechanism is, what its downstream effects are, and whether these contributes to bacterial killing later on. In this review we have tried to clarify these points by digging deep into the available literature on the effects of daptomycin on bacterial cells and can confidently say that simple pore formation is not the primary antibacterial mechanism of this lipopeptide.

It became clear that RIFs play a central role in the mechanism of daptomycin [54,64]. A structure as complex as this cannot be mimicked in in vitro model systems. Already the choice of simple two-lipid mixtures is a difficult one, since it can influence the behavior of the antibiotic [49,60,61,62]. It is therefore not surprising that in vitro results differ from observations in bacterial cells. Another factor is simply that prior to the extensive in vivo studies by Pogliano et al. and Müller et al. there was no reason to look for things like membrane fluidity or domain formation. After these publications however, in vitro studies were performed that confirmed these results: daptomycin’s preference for the fluid phase, its ability to form lipid domains, the transient nature of membrane permeability, and the threshold concentration needed for this all contribute to closing the gap between in vivo and in vitro observations [19,24].

## 10. Conclusions

Daptomycin is an important last resort antibiotic and one of the very few systemically-applied antibacterial drugs with a membrane-targeting mechanism of action. Although several resistant mutants were isolated, resistance development is still slower compared to drugs with single protein targets [163]. In contrast to well-characterized compounds like vancomycin, derivatives of daptomycin have not succeeded in making the transition into the clinic yet [12,164,165,166,167]. This may at least partly be attributed to our limited understanding of its mechanism of action. From the recent advances made in this field, we can learn important lessons for future drug development, not only for developing improved derivatives of daptomycin, but also for the design of novel lipopeptides and other membrane-targeting antibacterial drugs.
